# Potential Growth and Chemical Composition Changes During the Growth of New Zealand White Rabbits

**DOI:** 10.3390/ani15111670

**Published:** 2025-06-05

**Authors:** Adenike Adetutu Eniwaiye, Zikhona Theodora Rani-Kamwendo

**Affiliations:** School of Agricultural, Earth and Environmental Sciences, University of Kwazulu-Natal, Scottsville, Pietermaritzburg 3201, South Africa; raniz@ukzn.ac.za

**Keywords:** age, allometry, Gompertz curve, New Zealand White, rabbits, sex

## Abstract

This study was conducted on New Zealand White male and female rabbits over a period of 133 days to ascertain their potential growth rates, body composition for major body parts, and chemical makeup. A total of 220 New Zealand White rabbits, evenly distributed between males and females, were used for this study. One hundred rabbits for potential growth were weighed from day 14 to day 140, while twelve rabbits, six males and six females, were randomly selected at days 14, 21, 28, 35, 42, 56, 70, 84, 112, and 140 for carcass analysis. Although the rate of maturation was faster in females than in males, the Gompertz equation fitted separately to the growth data for males and females indicated that the body weights were similar throughout the trial (0.0243 vs. 0.0239), but males had a higher mature weight (315 g) than the females (309 g). Mature body protein weights averaged 1497 g in males and 843 g in females, and mature body lipid contents averaged 252 and 227 g, respectively. The rate of maturation per day of pelt-free body protein of males and females was 0.0103 and 0.0172, while that of body lipids was 0.0410 and 0.0471, respectively. Separate equations were required for males and females to describe the allometric relationship between protein and lipids in the pelt-free body. The rate of maturation of pelts in females was higher than in males (0.0249 vs. 0.0214/d), and the mature weight was lower (456 vs. 523 g, respectively).

## Simple Summary

This study examined how New Zealand White rabbits grow and how there are changes in their body composition over 133 days. This study utilized 220 rabbits, with an equal number of males and females, and then measured their weight before analyzing the chemical makeup of their body parts. At first, males and females grew at similar rates, but in the end, the males became heavier than the females, reaching about 1766 g compared to 1754 g for females by day 140. Also, the males had more body protein and fat than the females. But the females matured faster, especially in terms of body protein and fat. This study found that there is a relationship between body protein and other components, like fat and water, which are not the same between males and females. These findings help farmers and scientists understand how rabbits grow, which can improve breeding and feeding practices to produce healthier rabbits and better meat products.

## 1. Introduction

Because rabbits have historically been chosen for growth, a lot of attention has been paid to their slaughter weight. Currently, a broad variety of rabbit body parts are utilized, providing a range of products from the entire carcass to its component parts. Growth simulation, which is provided by slaughter maturation, benefits greatly from growth analysis. This study builds on previous research, such as Fodor et al. [1] and Dal Bosco et al. [2], by examining a longer growth period and the detailed chemical composition of New Zealand White rabbits. Male and female turkeys undergo different stages of slaughter development, according to Emmans [3]. The potential growth rate of an animal refers to its maximum growth without being constrained by other factors [4,5]. When animals are grown under non-limiting conditions, potential growth can be observed, which is assumed to reflect the genotype of the animal. Growth is supported by the development of internal organs. Tumova and Chodova [6] observed that in restricted chickens, priority is given to the growth of internal organs compared to muscles. Weight gain in the body is typically accompanied by weight gain in the carcass and its component parts. Although these conditions cannot be precisely defined, experiments with broilers [7,8,9] and pigs [10] have demonstrated that suitable experiments can provide realistic estimates of potential growth parameters. At that point, the animal’s capacity for growth depends on its existing condition. According to Murawska et al. [11], as turkeys mature, their carcass composition changes in different sections of the carcass: the amount of muscle tissue rises, the amount of bone falls, and the amount of skin with subcutaneous fat stays constant. In this study, the Gompertz form was used to describe growth since it has been found to be a reliable and useful method of describing a growing animal’s potential. Growth simulation, using models like the Gompertz equation, enables the prediction of growth trajectories, aiding optimization of feeding and breeding strategies. Post-slaughter maturation data refine these simulations by providing insights into carcass composition changes, enhancing model accuracy. These points above show the importance of understanding the potential growth and carcass chemical composition of New Zealand White rabbits. This study was carried out to evaluate the allometry of the chemical composition of the carcasses of male and female New Zealand White rabbits from day 14 (2 weeks) to day 140 (20 weeks) of age. Therefore, this study aims to evaluate the allometry of the chemical composition in New Zealand White rabbits, providing insights into growth patterns that can optimize breeding and feeding strategies to enhance meat quality and production efficiency.

## 2. Materials and Methods

The experiment was approved by the Ethics Committee of the University of KwaZulu Natal, Pietermaritzburg, KwaZulu Natal, South Africa, with ethical clearance number AREC/00003293/2021. The experiment took place at the Ukulinga research farm, of the University of KwaZulu-Natal (UKZN), Pietermaritzburg, Republic of South Africa (RSA). A total of 220 New Zealand White rabbits, evenly distributed between males and females, were used in this investigation for a duration of 14 to 140 days. Male and female rabbits were raised separately in separate cages. A total of 120 rabbits were employed for the sampling process, and 100 were used to assess potential growth. Twelve rabbits, six males and six females, were randomly selected at each of the following time points for the purpose of carcass analysis: 14, 21, 28, 35, 42, 56, 70, 84, 112, and 140 days. The 100 rabbits for potential growth were weighed from day 14 to day 140. All rabbits were fed ad libitum with a commercial pelleted diet containing 16% crude protein, 3% fat, 14% fiber, and 10.5 MJ/kg metabolizable energy, manufactured by AFGRI Animal Feeds, Ohrtmann road, Pietermaritzburg, South Africa. The diet consisted of maize, soybean meal, alfalfa hay, and a vitamin–mineral premix, formulated to meet the nutritional requirements of growing rabbits. Rabbits were weaned at 28 days of age. In the pre-weaning period, kits had access to maternal milk and a starter diet (18% crude protein, 2.5% fat) from day 14, manufactured by AFGRI Animal Feeds, Ohrtmann road, Pietermaritzburg, South Africa. In the post-weaning period, they transitioned to the standard grower diet. Rabbits were housed individually in wire cages (60 × 40 × 35 cm) under controlled conditions (20–22 °C, 60–65% humidity, 12 h light–dark cycle).

All through the duration of the study, rabbits selected for potential growth assessment had their individual weights taken on a weekly basis, while the measurements of all the body components of the rabbits used for sampling were made as they were being slaughtered at the abattoir on the stipulated sampling days (days 14, 21, 28, 35, 42, 56, 70, 84, 112, and 140). The rabbits were electrically stunned at this facility before the slaughtering procedure was carried out by professional workers. Prior to the slaughtering process, each rabbit’s weight was measured to determine its live weight. Subsequently, after the slaughtering was completed, the weight of the rabbit without its pelt was recorded to obtain the pelt-free weight. The weights of the pelt, internal organs (the stomach, heart, kidney, and liver), and eviscerated body parts (the head, fore and rear limbs, ribs, and saddle) were also determined. The units of measurement were all in grams. The rabbits were suspended on a cutting hanger after the initial cut at the neck that followed electrical stunning on various slaughter days (14, 21, 28, 35, 42, 56, 70, 84, 112, and 140). The subsequent cut was typically made at the hind feet, running from one thigh to the other, and then the skin was carefully removed in a single piece. The feed intake and feed conversion ratio were not measured due to logistical constraints at the research facility, which lacked automated feeding systems. This limitation precludes direct assessment of feed efficiency, and future studies should include these measurements to fully evaluate growth potential.

The body parts in bags were subsequently placed in freezers until the mincing procedure was carried out. Carcasses were placed in labeled bags and frozen at a temperature of −4 °C for later processing. To create homogenous samples, each component was processed individually by a meat grinder after thawing. The moisture content of these samples was then determined by freeze-drying at a temperature of −50 °C using a Supermodulyo Freeze Dryer from Thermo Electron Corporation, Edwards, Asheville, NC, USA. The samples were again ground after being freeze-dried, but this time with an Ika A11 Basic coffee bean grinder from IKA-Werke GmbH & Co. KG, Staufen, Baden-Württemberg, Germany. Ash content was calculated as the differences between pelt-free body weight and the weight of water, protein, and lipids. Crude protein was calculated using the N LECO analyzer with the Dumas combustion technique using a known conversion factor of 6.25 (AOAC 992.15; AACC 46-30) from the nitrogen concentration, manufactured by LECO Corporation, St. Joseph, MI, USA. Crude fat content was determined as described in ISA 1444 (1997) [12] using petroleum ether as the extraction solvent, supplied by Merck KGaA, Darmstadt, Hesse, Germany, in a Soxtec 1043 apparatus from FOSS Tecator AB, Höganäs, Skåne, Sweden.

Differences in live weight, pelt-free weight, and chemical components (protein, water, lipids, ash) across ages and sexes were analyzed using ANOVA in GenStat 23rd edition software from VSN International, Hemel Hempstead, Hertfordshire, UK [13]. The 100 rabbits used for growth assessment were individually marked and weighed weekly. Growth data were analyzed using a mixed model with rabbit as a random effect to account for repeated measures. No rabbits were lost during the experiment. Linear regression was used to determine the relationship between body components and body proteins. The weights of the chemical components were converted to natural logarithms (ln) to determine their allometric relationships. No generative artificial intelligence was used in the preparation of this manuscript.

## 3. Results

### 3.1. Growth Analysis

Table 1 shows the average live weight values for each sex at various ages. Throughout the study, the growth rates of the two sexes were comparable, but after day 35, males began to grow considerably faster than females. Over the course of the weighing period, the data showed a progressive and linear increase in the live weight. The rabbits’ prospective growth rate was obtained by examining each individual’s body weight. At 140 days of age, the mean body weights attained by the two sexes were 1754 g for the females and 1766 g for the males; however, the males’ averages were substantially larger (*p* < 0.001). Significant differences were seen in the age–sex interaction (*p* < 0.003) and in sex and age (*p* < 0.001). The pelt-free males and females responded differently (*p* < 0.001) at all the growth stages, and there was a significant effect (*p* < 0.001) on age across both sexes. No significant effect of sex was found (*p* > 0.05), although males had numerically higher pelt-free weights (1146 g) than females (1098 g) (Table 2). The pelt-free weight increased linearly with age but not with sex. There was a linear increase in all relative body weights across the sampling ages. The mean weight of the males (155 g) was more than that of the females (147 g).

Table 3 shows that sex and age affect the fore and hind limbs. All growing ages showed different responses in the males and females for both body components. In the hind limbs, there was a significant age–sex interaction and a significant age effect (*p* < 0.001) on the age of the two body components for both sexes. There were clear variations in pelt weights between ages and age–sex interactions, with highly significant differences (*p* < 0.001) (Table 3). The weight of the females’ pelts was greater than the males’ on days 14 to 28. However, by day 35, the weight of males’ pelts had increased to 130 g, which was higher than that of females’ pelts (Table 3). The Gompertz growth curve fully described growth in the female (R^2^ > 0.980) and male (R^2^ > 0.990) New Zealand White rabbits, according to an analysis of the individual potential growth weight data (Table 4). Significant differences in the three parameters were found when fitting the Gompertz equation to these data for different age groups, requiring distinct equations for males and females. The mature weight was higher in males (47.4) than in females (46.7), and the age at maximum growth rate was also higher in males (315) than in females (309) (Table 4).

### 3.2. Chemical Composition of Pelt-Free Body

**Figure 1:**
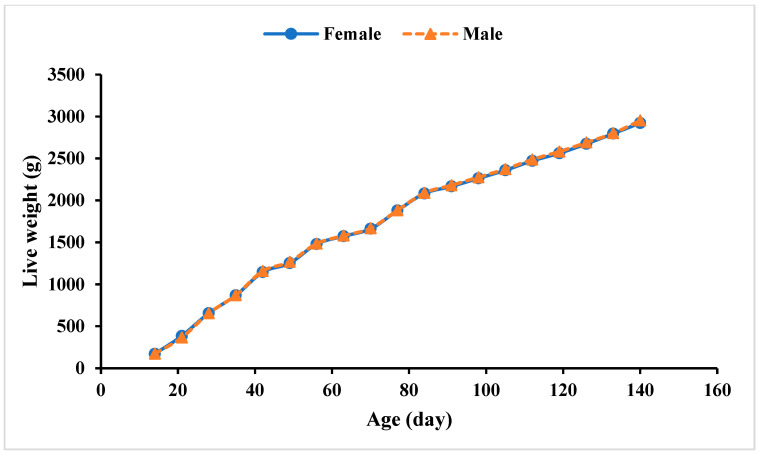
Growth curves of male and female NZW rabbits.

On average, a 14-day-old rabbit contained 819 g/kg water content, 103 g/kg protein, 54 g/kg lipid, and 25 g/kg ash (Table 5). The pelt-free body included the head, internal organs, fore and hind limbs, ribs, and saddle body components; the pelt was excluded. The interaction between sex and age was the primary factor affecting these results (*p* < 0.001). The males’ body protein content fluctuated during growth, declining at day 42, increasing at day 84, and then declining at day 112. Males experienced larger age-related changes in body lipid and protein contents than females did. There was no explanation for the observed inconsistency in body lipids. Throughout the growing period, the body’s water content decreased linearly with time; these trends persisted for both sexes. At days 14 and 21 for both sexes, the body ash content did not show any trend over time. From day 28 to day 70, it was more consistent, but from day 84 onward, it once again showed no trend. Table 6 shows the description of the growth of body components in the pelt-free body of male and female rabbits using the Gompertz growth curve. Females had a lower mature pelt weight of 45 g/kg compared to the males’ 52 g/kg, but their rate of pelt maturation (B) was significantly higher (0.02496 vs. 0.02135) than that of the males (Table 6). The growth curves of the male and female New Zealand White rabbits are displayed in Figure 1. When the live weight was plotted against age, the two sexes showed almost identical growth patterns on similar paths. To calculate the parameters of the Gompertz growth curves, the increasing weights of body protein in the pelt-free body over time were used. Table 7 lists these parameters for the body parts and sexes that were used in the experiment. Although the females’ mature size (74 g) was less than the males’ (123 g), the females’ protein matured at a faster rate than the males’ (0.017 vs. 0.010/d). The Gompertz equation (Table 7) predicted that the mature weight of water content would be heavier in males (34 g vs. 32 g, respectively), and that the females would mature at a faster rate (0.039 vs. 0.036/day, respectively). At maturity, the body lipid weights of the males and females were 42 g and 39 g, respectively, and the rates of maturation for these chemical components were also similar. Females had a lower age at maximum growth rate body protein weight (843 vs. 1497 g/kg) than males, but they matured at a higher rate (0.0172 vs. 0.0103/d) than males.

### 3.3. Allometric Relationships in Chemical Components of the Body

The body water and protein contents of the pelt-free body showed a strong allometric relationship (R^2^ = 0.964) over the ages and for both sexes. The equation describing this relationship is ln body water, g = 3.1714 ± 0.0592 + 0.6310 ± 0.0112ln body protein. Also, the equation used to describe the allometric relationship between body lipids and protein of the pelt-free body in males and females is ln body lipids, g = −0.7089 ± 0.0945 + 1.0041 ± 0.018ln body protein, with a strong allometric relationship of 0.963 existing between them. Sex-specific allometric equations for lipids vs. protein are as follows: males, ln body lipids, g = −0.7200 ± 0.0950 + 1.0100 ± 0.0190 ln body protein (R^2^ = 0.965); females, ln body lipids, g = −0.6978 ± 0.0940 + 0.9980 ± 0.0180 ln body protein (R^2^ = 0.960). ANCOVA showed no significant sex effect (*p* = 0.12). Ash content in the pelt-free body was allometrically related to body protein (R^2^ = 0.979), the equation being ln ash, g = −1.5281 ± 0.0730 + 1.0145 ± 0.0138*ln body protein. The weight of the ash content was numerically lower in the body than all other chemical components. The allometric relationship with ln body protein showed this shift over time, with the regression coefficient for ash being higher than that of lipids (1.0145 vs. 1.0041), but the constant term of the ash component was lower than that of lipid components (−1.5281 vs. −0.7089, respectively).

#### Equation Examples

This is Equation (1):

(1) ln Y=a+b In X 

This is Equation (2):

(2) ln Y body water, g=3.1714 ±0.0592+0.6310 ±0.0112×In body protein

## 4. Discussion

The experiment aimed to determine the growth rate and chemical composition of the carcasses of a few body parts in both male and female New Zealand White rabbits. It was crucial to make sure that the growth rates of the sexes and ages differed to accomplish this, particularly in terms of potential growth. In this regard, the experiment was successful because there were significant variations in the ages of all body parts that were measured. All of the physical components that were measured showed weight variations throughout all age groups (days), supporting previous findings indicating that these components seem to vary by sex [14]. The two sexes analyzed in the trial for growth and carcass analysis turned out to be nearly identical because they had comparable mature body weights and protein, water, and lipid contents, as well as remarkably similar maturation rates. While male and female NZW rabbits exhibited similar growth patterns, the significant sex–age interactions indicate differences in maturation rates and body composition. In contrast to the findings reported by Dal Bosco et al. [2] and Yalcin et al. [15], the mean values for live weights obtained in this study were higher in males than in females. These variations could have been caused by a number of variables, including feeding circumstances, weaning ages, environmental factors, and slaughtering ages [16], but they did not significantly affect the results of the current study (Table 1). Compared to the studies of Fodor et al. [1] and Dal Bosco et al. [2], which focused on shorter growth periods, our study’s 140-day duration and detailed chemical composition analysis provide a more comprehensive view of NZW rabbit growth and carcass development. The lower body weights observed (e.g., ~1.7 kg at 140 days) compared to typical NZW weights (~2.5 kg at 90 days) may reflect genetic variation in the strain or the absence of growth promoters in the diet. It has been noted that both sexes’ live weights increased as they aged. This is expected because, as the animal grows, its body size and shape should also increase in tandem with age until maturity, at which point growth will gradually slow down and eventually cease [17]. A proper description of potential growth will address the systematic changes in the physical composition of the body that occur during growth [8]. The intended result was a wide range of body growth rates and weights of the physical components of the animals in the sample, produced in the two sexes (male and female). In the current study, the sex of the rabbits did not seem to have any effect on the physical body components’ growth patterns. The body protein in the chemical composition of the carcass caused a significant difference (*p* < 0.001) in the response from both male and female NZW rabbits. Fluctuations in male protein and lipid contents, particularly at 84–112 days, may show the onset of puberty, which can alter lipid deposition, or sampling variation due to individual differences among rabbits. Geneticists could therefore benefit from the innate growth and developmental differences between the sexes by raising male and female rabbits apart while providing them with a similar nutritional diet. A balanced, non-limiting nutritional schedule improved growth performance, according to the study’s findings. Since nutrients can be adjusted to promote faster growth and higher body weights in males, as demonstrated in this trial, raising the sexes separately may help to increase body weight uniformity. As a formal way of comparing the potential growth rates of the sexes at maturity, it is interesting to determine how much the relationship between the various body components and body protein weight would differ between male and female NZW rabbits by fitting Gompertz growth curves to these data for the sexes. Therefore, statistical comparisons could be made between the three Gompertz equation components rather than mean weekly weights. These details can be used to characterize the breed [3]. Females matured earlier than males, as evidenced by their higher maturation rate (B = 0.02496 vs. 0.02135) and lower age at maximum growth rate (t* = 309 vs. 315 days). This is because the rate of maturation is inversely related to pelt-free body weights. Additionally, all males had higher mature weights than females in each body component measured at the end of the trial. This indicates that females matured at a faster rate than males, which led to their mature weight being higher earlier. The rate of maturation of protein in females (0.0172) was faster than in males (0.0103), while males had a higher mature weight than females (1497 vs. 843 g/kg). Also, for body water and body lipids, the mature weight of the males was heavier than that of the females (1260 vs. 1191 g/kg and 252 vs. 227 g/kg, respectively). In terms of the rate of maturation, body water and body lipids matured faster in females than in males (0.0391 vs. 0.0356 g/kg and 0.0471 vs. 0.0410, respectively). It is expected that body water, lipid, and ash contents in NZW rabbits growing to their genetic potential will be allometrically related to body protein [18]. This was found to be true in this experiment with the equations shown in Table 6. In order to determine allometric relationships between these chemical components and body protein, Riveira Torres et al. [19] related the weights of body protein, water, lipids, and ash to pelt-free body weight. Simple power functions offer good descriptions of these relationships. When two variables share the same rate of maturation, they are said to be allometrically related [20]. In the chemical composition of this study, the rates of maturation shared by the water, protein, and lipid contents were the same. The water deposition in the NZW rabbits was much higher than the protein deposition, according to allometric coefficients characterizing the correlations between the water, lipid, and ash weights and protein weight in this study. The amount of water in the body dropped as the age of the rabbits increased, although the proportion of protein increased, as shown by the allometric coefficients. While lipid deposition is influenced by dietary and environmental factors, protein deposition is controlled by genetic factors [21]. The growing percentage of body lipids is usually responsible for the decreasing rate of water deposition in relation to body weight throughout growth. According to the current findings and those of Gous et al. [8], variations in the fraction of tissues with varying water-to-protein ratios may be the cause of the decrease in water weight as compared to protein weight. The allometric relationship between body protein and live weight in this study demonstrated an isogonic trend, with protein rising in proportion to the rise in live weight.

## 5. Conclusions

Based on the results, while male and female NZW rabbits exhibit similar growth patterns, significant sex–age interactions indicate differences in maturation rates and body composition. Males have higher mature weights, while females mature faster, reaching their maximum growth rate earlier. Body protein contents correlate with physical body parts, and pelt-free body weight serves as a reliable indicator of chemical composition. Sex-specific feeding strategies may improve growth uniformity, but this hypothesis requires further research to confirm its efficacy. As a key indicator of body and carcass composition, pelt-free body weight can be used to estimate the chemical composition of NZW rabbits accurately. The findings regarding the ash, lipid, water, and protein contents of the pelt-free body are objective, making them useful for figuring out the chemical makeup of rabbits. With the help of this description of rabbit growth potential, it will be possible to predict rabbit growth models as well as the physical and chemical makeup of these rabbits’ growth more precisely. Therefore, it is concluded that male New Zealand White rabbits exhibit higher mature weights than females, while females mature faster. Body protein correlates with physical body parts, and pelt-free body weight reliably estimates chemical composition, aiding in predicting growth models for improved breeding and feeding practices.

## Figures and Tables

**Table 1:** Mean live weight (g) of male and female rabbits at different sampling ages.

Ages (Days)	Males	Females	Mean
14	173	172	173
21	367	386	376
28	655	657	656
35	871	867	869
42	1158	1146	1152
49	1269	1253	1261
56	1486	1480	1483
63	1581	1574	1577
70	1669	1663	1666
77	1883	1880	1882
84	2090	2084	2087
91	2181	2167	2174
98	2277	2263	2270
105	2374	2360	2367
112	2489	2472	2480
119	2582	2563	2573
126	2690	2675	2683
133	2801	2797	2799
140	2953	2869	2911
Mean	1766	1754	1760

*p* < 0.001: significant; age–sex: *p* < 0.003.

**Table 2:** Mean live, pelt-free, and head weights (g) of female and male rabbits at 10 sampling ages.

Live				Pelt-Free			Head		
Age (Days)	Female	Male	Mean	Female	Male	Mean	Female	Male	Mean
14	172	179	175	134	142	217	42	41	42
21	386	375	380	308	313	413	65	65	65
28	663	662	663	570	584	621	91	96	94
35	853	864	859	727	734	900	116	117	116
42	1148	193	1170	942	995	1175	130	140	135
56	1506	1461	1484	1252	1256	1375	151	155	153
70	1566	1513	1540	1275	1332	1442	157	159	158
84	2094	2132	2132	1778	1801	1795	210	220	215
112	2292	2392	2392	1924	2020	2324	242	270	256
140	2454	2749	2749	2067	2287	2541	268	283	276
Mean	1313	1352	1333	1098	1146	1280	147	155	151
	Age	Sex	Age*Sex	Age	Sex	Age*Sex	Age	Sex	Age*Sex
*p*-Value	***	NS	NS	***	NS	NS	***	NS	NS
SEM	51	22.8	7.1	44.65	19.97	63.15	6.32	2.82	8.93
RMS		15,613			11,964			239	

SEM: standard error of mean; RMS: residual mean square; F: female; M: male; NS: not significant. ***: significant.

**Table 3:** Mean pelt, fore limb, and hind limb weights (g) of female and male rabbits at 10 sampling ages.

Pelt				Fore			Hind		
Age (Days)	Female	Male	Mean	Female	Male	Mean	Female	Male	Mean
14	38	37	38	42	41	42	38	37	38
21	73	71	72	65	65	65	73	71	72
28	93	78	85	91	96	94	93	78	85
35	126	130	128	116	117	116	126	130	128
42	198	206	202	130	140	135	206	198	202
56	205	224	215	151	155	153	224	205	215
70	234	238	236	157	159	158	234	238	236
84	316	331	324	210	220	215	316	331	324
112	367	373	370	242	270	256	367	373	370
140	387	463	425	268	283	276	387	463	425
**Mean**	**204**	**215**	**210**	**147**	**155**	**151**	**206**	**212**	**210**
	Age	Sex	Age*Sex	Age	Sex	Age*Sex	Age	Sex	Age*Sex
*p*-Value	***	NS	NS	***	NS	NS	***	NS	NS
SEM	10.83	4.85	15.32	6.32	2.82	8.93	10.83	4.85	15.32
RMS		704			239			704	

SEM: standard error of mean; RMS: residual mean square; F: female; M: male; NS: not significant, ***: significant.

**Table 4:** Gompertz parameters describing the potential growth of male and female New Zealand White rabbits. The Gompertz equation is of the form A + C × exp(−exp(−B × (X−M))), where A is the asymptotic weight (g), C is the amplitude of growth (g), B is the maturation rate (per day), M is the age at maximum growth rate (days), and X is age (days).

Parameter	Male	Female	SEM	*p*-Value
A(g)	47.4	46.7	0.5	<0.05
B (g/d)	0.0243	0.0239	0.0002	NS
C (g/kg)	315	309	3.2	<0.05
M (days)	315	309	2.8	<0.05

SEM: standard error of mean; NS: not significant (*p* > 0.05).

**Table 5:** Mean pelt-free body protein, lipid, water, and ash contents (g/kg) of male and female New Zealand White rabbits at different ages.

**Age (Days)**	**Sex**	**Protein (g/kg)**	**Lipid (g/kg)**	**Water (g/kg)**	**Ash (g/kg)**
14	M	102	49	828	22
14	F	104	58	810	28
21	M	123	56	799	22
21	F	124	44	808	23
28	M	169	68	719	44
28	F	161	65	733	41
35	M	193	90	675	42
35	F	180	93	687	41
42	M	189	89	672	50
42	F	184	102	673	42
56	M	196	149	599	56
56	F	202	156	603	44
70	M	200	110	643	46
70	F	211	105	636	48
84	M	207	130	628	36
84	F	224	88	648	40
112	M	194	93	661	52
112	F	258	105	574	63
140	M	299	126	493	82
140	F	292	112	523	73
SEM		2.54	4.50	11.38	1.81
*p*-Value	Age	<0.001	<0.001	<0.001	<0.001
	Sex	NS	NS	NS	NS
	Age–sex	<0.001	<0.001	<0.001	<0.001

*p* < 0.001: significant; SEM: standard error of mean; NS: not significant; M: male; F: female.

**Table 6:** Parameters of the Gompertz growth curve (age at maximum growth rate, rate of maturation, and mature weight) ± SE, describing the growth of body components in the pelt-free body of male (M) and female (F) New Zealand White rabbits.

	Age at Maximum Rate, t*, d		Rate of Maturation,/d		Mature Weight, g/kg		
Item	Mean	SE	Mean	SE	Mean	SE	R^2^
Pelt-Free							
Males	2387	90.8	0.0278	0.0024	42.71	2.02	0.95
Females	2229	49.9	0.0309	0.0018	39.93	1.13	0.96
Pelt							
Males	522.5	36.5	0.0214	0.0026	52.19	4.29	0.93
Females	456.4	17.3	0.0249	0.0019	44.80	2.11	0.93
Head							
Males	325.6	19.4	0.0209	0.0025	41.45	3.55	0.93
Females	311.8	12.5	0.0209	0.0017	39.65	2.35	0.93
Forelimbs							
Males	296.7	18.7	0.2425	0.0032	43.10	3.53	0.91
Females	273.0	10.5	0.2626	0.0023	39.46	2.04	0.91
Hindlimbs							
Males	521.1	31.1	0.0282	0.0039	41.08	3.12	0.88
Female	497.0	17.1	0.0313	0.0028	38.83	1.73	0.99
Organs							
Males	131.3	1.97	0.0671	0.0044	28.32	0.624	0.97
Females	128.7	1.48	0.0673	0.0034	27.81	0.479	0.96

**Table 7:** Gompertz parameters (age at maximum growth rate, rate of maturation, and mature weight), describing the growth of the body chemical components (protein, water, and lipids) in the pelt-free body of male and female New Zealand White rabbits.

	Age at Maximum Rate, t*, d		Rate of Maturation,/d		Mature Weight, g/kg		
Parameter	Mean	SE	Mean	SE	Mean	SE	R^2^
Body protein							
Male	1497	569	0.0103	0.0028	123.4	37.0	0.91
Female	843	73	0.0172	0.0018	73.54	6.17	0.93
Body water							
Male	1260.0	44.8	0.0356	0.0038	33.71	1.71	0.92
Female	1191.3	25.1	0.0391	0.0026	31.92	0.99	0.93
Body lipids							
Male	252.1	13.3	0.0410	0.0062	41.85	2.50	0.86
Female	226.8	7.78	0.0471	0.0052	38.78	1.56	0.85

## Data Availability

The data presented in this study are available on request from the corresponding author. The data are not publicly available due to institutional restrictions.

## Abbreviations

The following abbreviations are used in this manuscript:

NZW	New Zealand White
SEM	Standard Error of Mean
RSD	Residual Mean Square
SE	Standard Error
ANOVA	Analysis of Variance
